# Sodium iodide modified-red mud for efficient adsorptive removal of methylene blue from wastewater: isotherm modeling and adsorption

**DOI:** 10.1039/d5ra06455d

**Published:** 2025-10-24

**Authors:** Muhammad Sarfraz, Farishta Shafiq, Karma M. Albalawi, Nadeem Raza, Ibrahim A. Shaaban, Asim Waseem, Irfan Ijaz

**Affiliations:** a State Key Laboratory of Fine Chemicals, School of Chemical Engineering, Dalian University of Technology Dalian 116024 P. R. China; b Department of Chemistry, Faculty of Science, University of Tabuk Tabuk Saudi Arabia; c Department of Chemistry, College of Science, Imam Mohammad Ibn Saud Islamic University (IMSIU) Riyadh Kingdom of Saudi Arabia; d Research Center for Advanced Materials Science (RCAMS), Chemistry Department, Faculty of Science, King Khalid University P. O. Box 9004 Abha 61413 Saudi Arabia; e School of Chemistry, Faculty of Basic Sciences and Mathematics, Minhaj University Lahore Lahore 54700 Pakistan iffichemixt266@gmail.com

## Abstract

The discharge of synthetic dyes and the improper disposal of industrial red mud (RM) pose serious environmental challenges worldwide. This study introduces an innovative adsorbent, RMI, synthesized by functionalizing raw red mud with sodium iodide (NaI) to remove methylene blue (MB) from aqueous solutions effectively. Comprehensive characterization using XRD, TG, DTG, SEM, and BET analysis confirmed the enhanced structural and surface properties of the RMI composites. Batch adsorption experiments revealed that RMI-5 achieved a maximum MB removal capacity of 245.2 mg g^−1^ under optimal conditions. The adsorption process followed pseudo-second-order kinetics (*R*^2^ = 0.99), suggesting that chemisorption is the dominant mechanism, while the Langmuir isotherm model best described the equilibrium behaviour. Thermodynamic analysis showed that MB adsorption was spontaneous and exothermic. The primary adsorption mechanisms include hydrogen bonding, electrostatic attraction, and pore diffusion. These findings demonstrate the high efficiency and sustainability of NaI-modified red mud as a low-cost, high-performance adsorbent for dye-contaminated wastewater treatment. Hence, this study illustrates the efficient elimination of contaminants, particularly methylene blue, from aqueous solutions with NaI-modified red mud composites. The results underscore the potential for practical applications in water treatment technologies for dye elimination and other pollutant remediation techniques.

## Abstract 2

The discharge of synthetic dyes and the improper disposal of industrial red mud (RM) pose serious environmental challenges worldwide.

## Graphical abstract

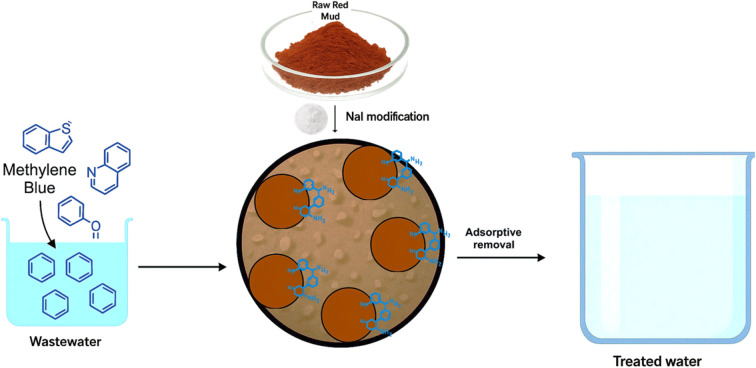

## 1. Introduction

The existence of life on earth is dependent on plentiful supplies of pure water, which is also responsible for the preservation of ecosystems through the development of agriculture, industry, and economic viability.^1,2^ Besides the fact that 71% of the earth's surface is covered by water, less than 1% is accessible for human consumption.^3^ Extensive industrialization and uncontrolled population growth result in water scarcity and water pollution due to the inclusion of diverse inorganic (metals and their salts) and organic (dyes, plastics, pharmaceuticals, petrochemicals, and pesticides) compounds.^4–6^ The drainage of untreated water from domestic and industrial sources contaminates aquatic systems, making them unsafe for humans and the environment, leading to severe concerns for the ecosystem.^7^ Among the diverse pollutant dyes released from different industries, including textile dyeing, paper, leather, and plastics, water-soluble cationic dyes severely contaminate water bodies.^8^ Currently, about 700 000 tonnes of textile dyes are used in the aforementioned industries, most of which are teratogenic, recalcitrant, and carcinogenic in nature.^9^ Further, the higher solubilities of dyes in aqueous media make them difficult to remove, which is why eliminating these pollutants has become one of the greatest challenges.^10^

The three major dye groups are believed to be cationic, anionic, and nonionic (water-insoluble) and are classified according to the charge of the chromophore group dissolved in water.^11^ The most often used cationic dye in the dyeing and ink production sectors is methylene blue (MB), which is environmentally hazardous due to its high water solubility and color resistance.^12^ When the residual dye from wastewater is discharged into water, even at low concentrations, it can harm aquatic life and humans in addition to causing sensory loss.^13^ MB prevents sunlight from entering water, disrupts the food chain, and leads to low oxygen levels.^14^ It is also toxic to cells, can damage DNA, lasts a long time, and does not break down naturally.^15^ Consequently, effective wastewater treatment and pollution control are desirable to preserve clean and sustainable water resources worldwide through the development of efficient and sustainable pollutant (MB) removal strategies.^16,17^

Conventional wastewater treatment methods, such as chemical precipitation, advanced oxidation processes and adsorption, have been explored to remove MB. Nowadays, adsorption is a popular technique for removing MB from wastewater due to its ease of use, low cost, environmental friendliness, and potential efficacy.^18^ Nonetheless, the adsorption approach for the efficient removal of target pollutants is still in its early stages. It faces several constraints in terms of installation costs, sustainability, regenerability, and environmental friendliness before commercialization. Their shape largely determines the adsorption capacity of the adsorbent particles. By altering the interaction between the adsorbate (contaminant) and the adsorbent, characteristics including surface area, porosity, shape, and particle size directly impact the effectiveness of adsorption. Over the past several years, extensive research has been dedicated to the development of novel adsorbents for the effective removal of MB from wastewater.

Adsorption with modified red mud (RM) is one of the most effective removal techniques necessary to reduce its adverse effects and maintain the water's purity.^19^ Recently, numerous studies have focused on the potential applications of red mud, which is also referred to as “high volume low effect waste”.^20^ It has several applications, especially in the fields of the cement industry, building and road surface material, and in the formation of bricks.^21^ According to reports, 4 to 5 tons of red mud, the primary waste product from extracting alumina from bauxite, are discarded for every ton of aluminium produced.^22^ The high surface area and rich content of iron oxides, alumina, and silica in this waste make it a promising low-cost adsorbent. Unfortunately, its low stability and adsorption capability frequently restrict its direct application. In order to get around these obstacles, modification methods, including heat treatment, acid activation, or functional ingredient addition, have been developed to improve its adsorption capabilities.^23^ Notably, layered and/or porous structures, such as modified red mud and clays with optimized particle morphology, high surface area and porosity, exhibit better pollutant diffusion, resulting in enhanced adsorption efficiency for targets.

Modification of RM through doping or combining it with other materials, like carbon foam, metal halides, or oxalic acid, can potentially improve its surface characteristics, such as increased active sites, and the synergistic effects of dopants and red mud make it highly effective in wastewater treatment and environmental remediation strategies.^24^ RM modification with metal halides, including NaCl, NaBr, and NaI, could result in improved surface roughness and pore size distributions along with increased negative charge, improved chemical and thermal stability, and the creation of new functionalities (such as M–O and X-related groups) on RM.^25^ The surface negative charge and new functionalities on RM could enhance hydrogen bonding, π–π interactions, and van der Waals forces, allowing for strong interactions with cationic targets (MB).

**Scheme 1:**
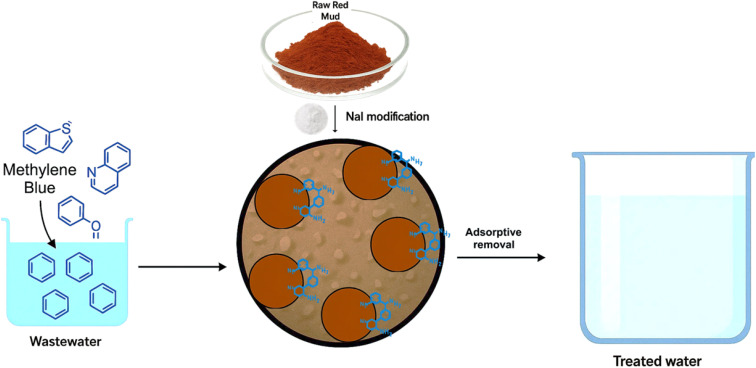
Illustration of the removal of MB using the RMI-5 composite.

In this study, a simplified method was developed to synthesize an efficient adsorbent by modifying the raw red mud. The synthesized material was extensively characterized to elucidate its physicochemical properties. Impregnation with NaI significantly enhanced the specific surface area and increased the availability of active adsorption sites, as shown in Scheme 1. The adsorption behaviour of the RMI-5 composite toward methylene blue (MB) was systematically investigated, with particular attention to key operational parameters, such as contact time, temperature, and initial dye concentration. Kinetic and isotherm models were applied to interpret the adsorption process and identify the dominant mechanisms, which were found to include hydrogen bonding, electrostatic interactions, and pore diffusion. These findings demonstrate the potential of RMI-5 as a cost-effective and sustainable adsorbent for dye removal from aqueous environments.

## 2. Experimental

### 2.1. Materials and methods

The red mud (RM), sodium chloride (NaCl), sodium bromide (NaBr), methylene blue (MB), sodium iodide (NaI), and sodium hydroxide (NaOH) used in this study were purchased from Aladdin Industrial Corporation, Shanghai, China. The structure of methylene blue is illustrated in Fig. S1. All the chemical reagents mentioned above were of analytical grade and used directly without additional purification.

### 2.2. Preparation of sodium halide-modified red mud

**Fig. 1:**
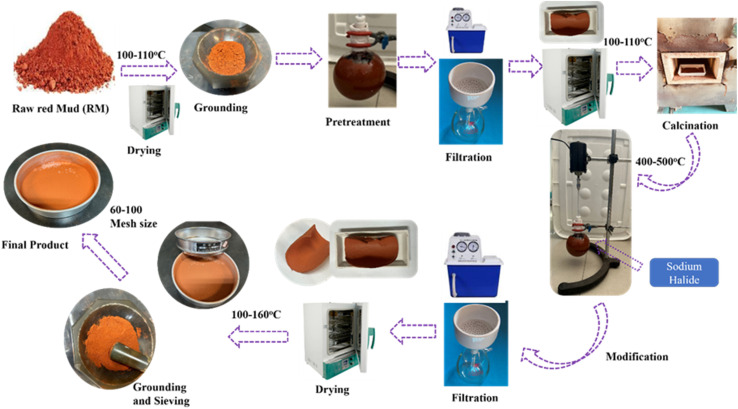
Schematic of the RM modification through sodium halide impregnation.

Raw red mud (RRM) was initially crushed and subsequently filtered *via* a sieve with an average mesh size smaller than 60. The resultant powder was dried in an oven for 24 hours at 110 °C to eliminate any residual moisture. Thereafter, the RRM was heated in a furnace at temperatures between 400 °C and 500 °C to break down the organic matter and improve its characteristics, including porosity and surface area. Sodium halide-functionalized raw red mud was synthesized using an impregnation strategy by adding a specific amount of NaI (3–5%) to 100 mL of DI water. Subsequently, 15 g of raw red mud powder was added to each sodium iodide solution and stirred consistently at room temperature for 6 hours. The resultant mixtures were subsequently dried for 20 hours at 110 °C. The NaI-functionalized RM sample was subsequently crushed to a particle size of less than 60 mesh, as illustrated in Fig. 1. The functionalized samples were labelled RMI-1, RMI-3, and RMI-5, representing red mud functionalized with 1%, 3%, and 5% sodium iodide, respectively.

### 2.3. Characterizations

The surface morphology images of RRMI, RMI-1, RMI-3, and RMI-5 were assessed using SEM (HITACHI-SU8220, Japan). X-ray diffraction (XRD) analysis of the RRMI, RMI-1, RMI-3, and RMI-5 composites was conducted using a Rigaku diffractometer (Japan). The pore volume and specific surface area of the as-prepared samples were calculated using the Brunauer–Emmett–Teller (BET) method (JW-BK200A, China). All adsorption experiments of the composites for MB were performed using a UV-visible spectrophotometer (Carry-100, Malaysia). The pH measurements were conducted using a PHS-3C pH meter with an integrated temperature sensor for precise temperature adjustment and regulation.

### 2.4. Batch adsorption experiments

Adsorption analyses were conducted in batch mode to evaluate the efficacy of the RMI-5 composite in removing MB dye. 25 mg of RMI-5 adsorbent was poured into a 150 mL beaker containing 60 mL of the methyl blue solution. The resultant mixtures were subsequently placed in a water bath shaker and agitated at 400 rpm for 40 minutes. The residual amounts of MB were quantified using a UV-vis spectrophotometer (Carry-100, Malaysia). The effects of key variables, including initial concentration, contact time, pH, and temperature, were systematically examined. The solution pH (2, 4, 6, 8, and 10) was adjusted using buffers, and their composition is listed in Table S1. All the experiments were performed in a triplicate, and the data were presented as a means to ensure reproducibility. A kinetic analysis was conducted by combining 25 mg of RMI-5 composite with 25 mL of MB solution to examine the removal quantities as a function of time. The adsorption isotherm was studied through several batch adsorption tests using 25 mg of RMI-5 composite and varying dosages of methylene blue at 298.15 K. Each test was conducted three times, and the means were calculated. Eqn (1) and (2) were utilised to compute the adsorption capacity and removal rate, respectively.1
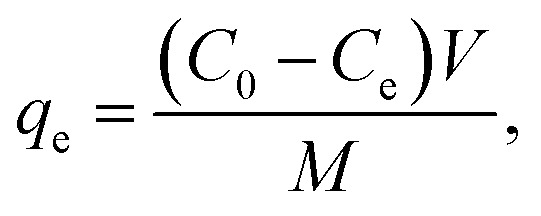
2
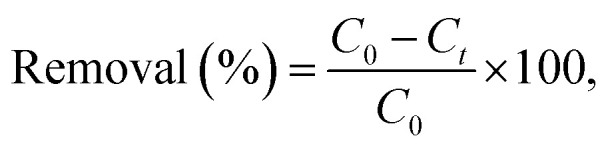
where *C*_e_ represents the equilibrium amount of MB (mg L^−1^) and *C*_0_ denotes the initial amount of MB (mg L^−1^). *M* and *V* denote the mass (mg) and volume (mL), respectively.

## 3. Results and discussion

### 3.1. Characterizations of raw and modified RM

In the present work, functionalized RM was prepared using sodium halide impregnation, as described in the Methods section. To thoroughly investigate the morphological characteristics, chemical composition, thermal stability, and surface porosity features of RRM and RMI (RMI-1, RMI-3, and RMI-5) particles, different instrumental techniques involving SEM, FTIR, XRD, TG, DTG, and BET were employed.

**Fig. 2:**
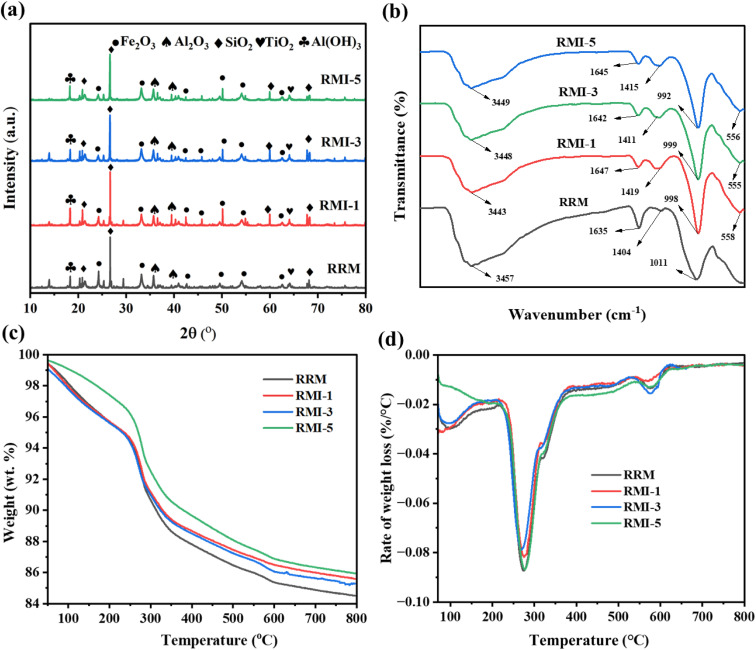
(a) XRD spectra of the raw and modified RM, (b) FTIR spectra of the raw and modified RM, (c) TG curve of the raw and modified RM, and (d) DTG curve of the raw and modified RM.

Fig. 2a illustrates the XRD spectra of the RRM, RMI-1, RMI-3, and RMI-5 adsorbents. The principal metal oxides, such as SiO_2_, Al_2_O_3_, and Fe_2_O_3_, were identified in both the unaltered RRM and modified (RMI-1, RMI-3, and RMI-5) materials, with their diffraction signal intensities remaining comparatively stable, indicating that the incorporation of NaI has a minimal impact on the structural composition of the raw red mud. No identifiable diffraction peaks for sodium iodide were detected in the RMI-1, RMI-3, and RMI-5 composites, indicating a uniform distribution of NaI over the surface.^26,27^ The FTIR spectra of RM and MRM were examined throughout the wavelength range of 500–4000 cm^−1^. The findings are presented in Fig. 2b. Fig. 2b illustrates that the diffraction peaks of each functional group transformed with the incorporation of NaI, with the appearance of a new peak. Prominent and extensive absorption bands were identified at 3457, 3434, 3448, and 3449 cm^−1^ for RRM, RM-1, RM-3, and RM-5, respectively, corresponding to OH stretching vibrations.^28^ Distinct peaks appearing at nearly 1635, 1647, 1642, and 1645 cm^−1^ for RRM, RMI-1, RMI-3, and RMI-5, respectively, signify the bending vibration of water, indicating a substantial intake of moisture from the surroundings.^29^ Peaks at 1404, 1419, 1411, and 1415 cm^−1^ observed in RRM, RMI-1, RMI-3, and RMI-5, respectively, are ascribed to (CO_3_)^2−^, indicating that RM comprises numerous carbonate molecules. The O–Si–O group appeared in the RRM, RMI-1, RMI-3, and RMI-5 samples, as evidenced by the appearance of peaks at 1011, 998, 999, and 992 cm^−1^, respectively. New peaks appeared at 558, 555, and 556 cm^−1^ in the FTIR spectra of RMI-1, RMI-3, and RMI-5, respectively, corresponding to the C–I groups. The appearance of C–I peaking suggests the successful introduction of sodium iodide.

Fig. 2c depicts the thermogravimetric evaluation of the RM samples based on the assessment of mass loss due to thermal exposure. Different weight loss levels were identified in the TG plots of the RMI-1, RMI-3, and RMI-5 composites. The findings suggest that the modified red raw mud demonstrated a slightly elevated water content compared to pristine red mud. Furthermore, all TG plots indicate three distinct phases of weight reduction occurring across various temperature ranges. The result suggests an early weight reduction of nearly 4% at 198 °C, which is due to the evaporation of adsorbed water. During thermal treatment, minerals such as ilmenite, goethite, and gibbsite undergo breakdown at temperatures ranging from 200 °C to 600 °C, resulting in approximately 14% weight loss. The weight reduction above 600 °C resulted from the breakdown of katoite only.

Fig. 2d depicts the DTG curves of the RRM, RMI-1, RMI-3, and RMI-5 composites. The highest rate of weight loss for RRM was observed at nearly 300 °C. The DTG investigation distinctly illustrates varying weight reduction trends for RRM, RMI-3, and RMI-5. The principal cause of the highest weight loss rate at approximately 300 °C in raw red mud is the decomposition of organic components (additives, hydrocarbons, and plant waste) and the release of volatile molecules and water that are physically entrapped within the raw red mud.^30^ The greater percentage of weight reduction noted at approximately 610 °C for RMI-1, RMI-3, and RMI-5 can be ascribed to the decomposition of NaI contained within the RMI. Halides alter the thermal durability of the substance by promoting the breakdown of complicated molecules at elevated temperatures. This illustrates the unique thermal properties of the modified sample.

**Fig. 3:**
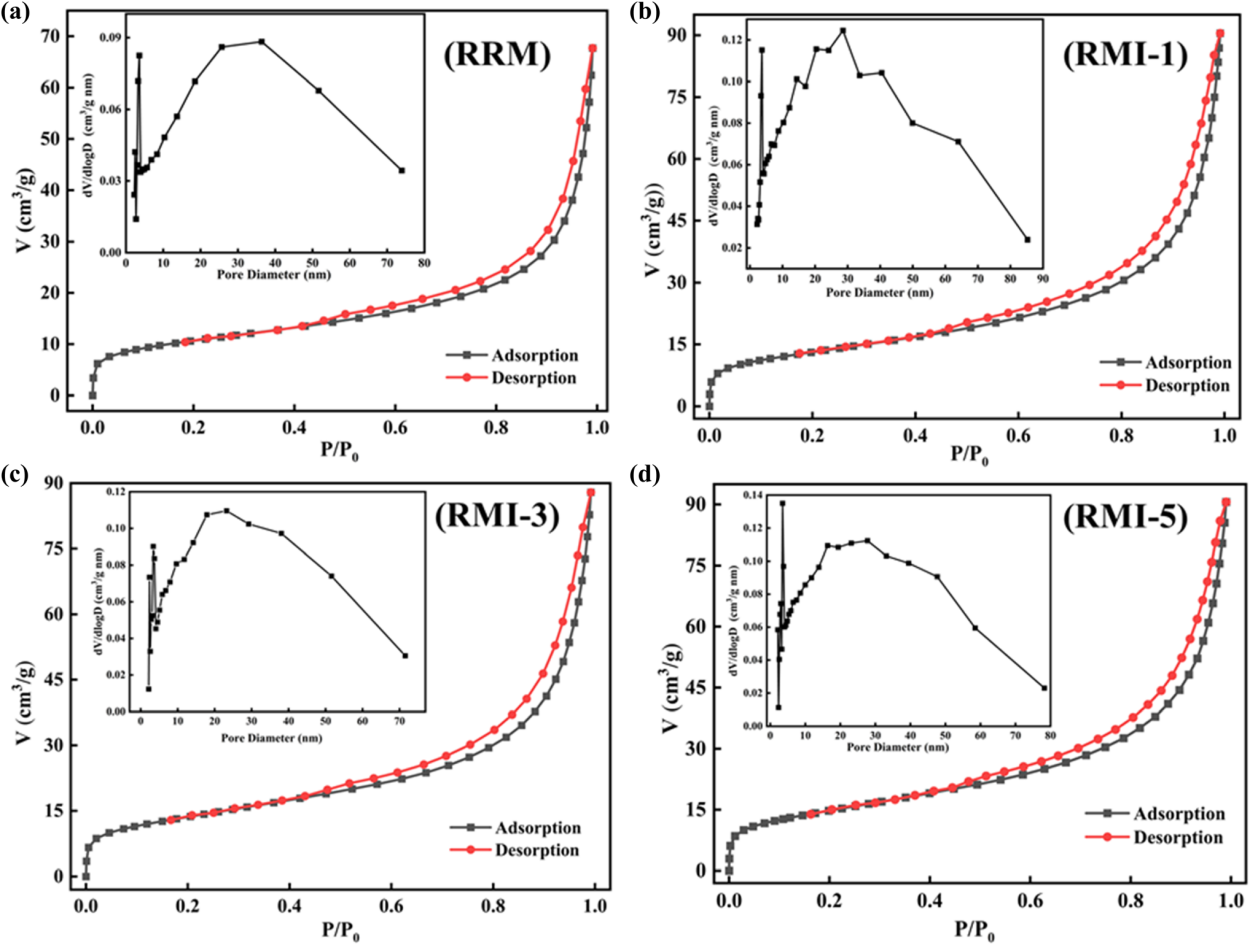
N_2_ adsorption–desorption isotherms and pore size distribution of (a) RRM, (b) RMI-1, (c) RMI-3, and (d) RMI-5.

The pore diameters, pore volumes, and specific surface areas (SSAs) of RRM, RMI-1, RMI-3, and RMI-5 are presented in Table 1. The SSA of the RRM was determined to be 37.4 cm^2^ g^−1^. Comparatively, the SSAs of the raw red mud particles subjected to 1%, 3%, and 5% sodium iodide were dramatically greater, measuring 46.8, 48.5, and 68.1 cm^2^ g^−1^, respectively. The substantial increase in SSA indicates that NaI loading enhances the surface characteristics of the raw red mud, with the 5% NaI treatment leading to the highest SSA. Based on the IUPAC classification, the isotherms of the RMI-1, RMI-3, and RMI-5 composites conform to type IV, exhibiting a distinctive H3 hysteresis loop (Fig. 3a–d). This behavior results from the formation of particle aggregates that form slit-like pores, demonstrating a characteristic mesoporous morphology. The higher pore diameter, pore volume, and SSA of RMI-5 significantly enhance its adsorption efficacy for MB. A higher SSA provides additional adsorption sites for MB to bind with the RMI-5 adsorbent surface, improving the uptake capability. A larger pore volume enhances the transport and retention of the MB dye within the RMI's internal structure. The existence of mesopores, often characterized by pore widths between 2 and 50 nm, is especially advantageous for the adsorption of MB owing to its molecular structure and size. Thus, RMI-5 was selected as the adsorbent for the subsequent adsorption of MB.

**Table 1:** N_2_ adsorption–desorption data of the raw and modified RM

Components	Specific surface area (m^2^ g^−1^)	Pore volume (cm^3^ g^−1^)	Average pore size (nm)
RRM	37.4	0.114	12.4
RMI-1	46.8	0.136	11.5
RMI-3	48.5	0.139	10.7
RMI-5	68.1	0.141	8.51

**Fig. 4:**
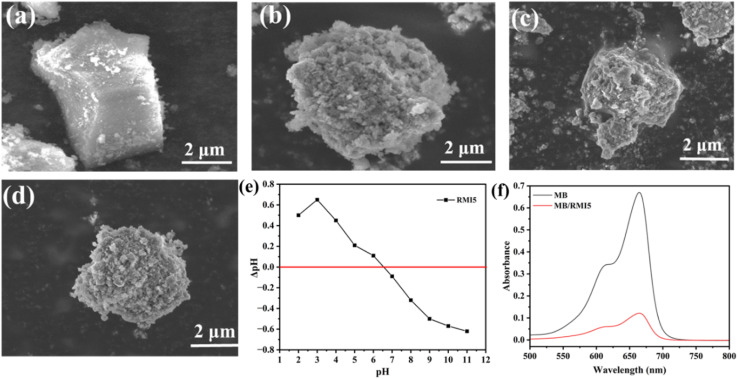
SEM images of (a) raw RRM, (b) RMI-1, (c) RMI-3, (d) RMI-5, (e) PZC determination of RMI-5 (point of zero charge = 6.5), and (f) the interaction of MB with the RMI-5 particles.

The surface morphological properties of RRM, RRM-1, RRM-3, and RRM-5 were studied using scanning electron microscopy (SEM). Fig. 4a depicts that the raw red mud possesses a comparatively smooth surface morphology. Fig. 4b–d exhibits the appearance of small aggregated particles on the smooth surface of raw red mud. The appearance of small particles justified the successful loading of the NaI particles. Furthermore, SEM images show increased porosity and surface roughness of the raw red mud after NaI impregnation, resulting in more pore channels and adsorption sites on the surface, which boosts the adsorption efficiency of MRM. The findings are consistent with the N_2_ adsorption–desorption studies in Table 1, indicating that the incorporation of NaI substantially influences the pore morphology of the impregnated materials.

### 3.2. Adsorption behaviour of modified RM

The point of zero charge (PZC) is regarded as one of the methods used to determine the nature of the charge on the surface of the adsorbent. It defines the pH value at which the adsorbent's overall surface charge density is zero. Thus, the PZC value for RMI-5 was determined. Fig. 4e illustrates that the point of zero charge (PZC) of RMI-5 was 6.5. This indicates that at pH levels exceeding the PZC, the surface of the RMI-5 possesses a negative charge, while at pH levels below the PZC, it acquires a positive charge. Consequently, at pH levels exceeding 6.5, the RMI-5 surface acquires a negative charge, which increases the electrostatic attraction between the cationic methylene blue (MB) and the negatively charged adsorbent. The adsorption performance is considerably enhanced at alkaline pH values. A spectroscopic approach was employed to investigate the dye adsorption properties at the contact point of the RMI-5 particles. The linearity of the spectrophotometric response for the dye was assessed by determining absorbance over suitable concentrations and constructing a calibration curve. A graph of concentration against absorbance was developed to confirm that methylene blue's absorbance corresponds to the Lambert–Beer law, as depicted in Fig. S2. Fig. 4f illustrates that the absorbance level of the methylene blue dye at its corresponding maximum wavelength (*λ*_max_) diminished upon interaction with the adsorbent, signifying the successful adsorption of the methylene blue dye on the negatively charged adsorbent.

#### 3.2.1. Effect of pH

**Fig. 5:**
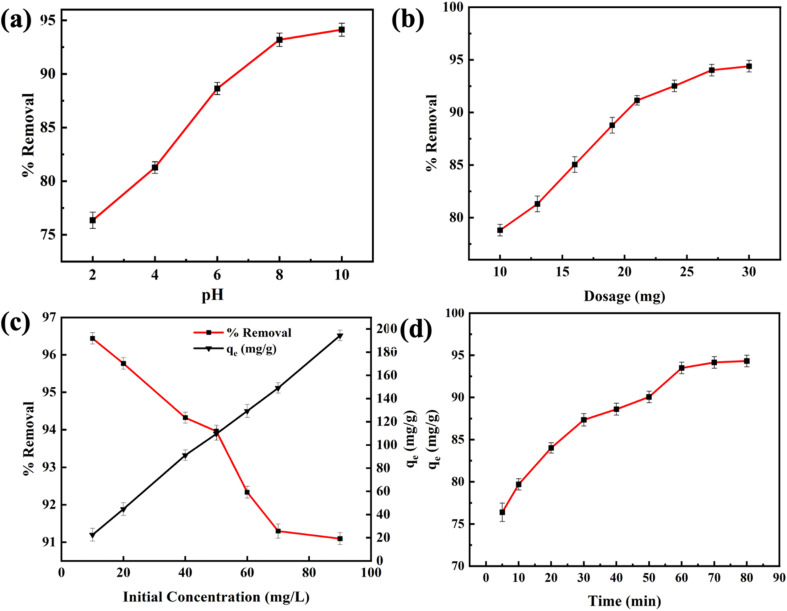
Factors affecting the adsorption of MB on the RMI-5 adsorbent: (a) impact of pH, (b) influence of adsorbent dosages, (c) effect of initial concentration of MB, and (d) contact time.

The pH is a critical parameter in the adsorption of pollutants, as it can affect the distribution of contaminants and the surface charge of adsorbents. Fig. 5a illustrates that RMI-5 exhibited high removal efficiency across the pH spectrum of 2–12. Under acidic conditions (pH less than 6.8), the removal efficiency of M-GNS was nearly 90%. The removal efficiency further improved with increasing pH, achieving 95% when the pH ≥ 6.8. The pH of the solution can influence the adsorbent's surface charge, which can be evaluated by the point of zero charge (pH_PZC_). The pH_PZC_ of RMI-5 was determined to be 6.8, as shown in Fig. 5a. When the pH was less than the pH_PZC_, the surface charge of RMI-5 was positive. Consequently, under acidic conditions, protons compete with methylene blue for active sites on the adsorbent surface, rapidly filling efficient active sites and leading to a slightly decreased removal rate. Conversely, in an alkaline environment (pH above pH_PZC_), the surface charge of RMI-5 is negative, which facilitates substantial electrostatic adsorption between the positively charged methylene blue and negatively charged adsorbent, resulting in a comparatively increased removal rate.

#### 3.2.2. Effect of adsorbent dosage

The adsorbent dosage is a crucial factor in methylene blue adsorption. The impact of varying dosages on MB elimination was examined, with the results illustrated in Fig. 5b. As the amount of adsorbent increased from 10 mg L^−1^ to 27 mg L^−1^, the removal performance increased from 78% to 94%, attributable to the accessible active sites for MB removal. Furthermore, an increase in adsorbent dosage may result in reduced removal efficiency. The reduction in efficiency may result from the overlap or aggregation of accessible adsorption sites at elevated adsorbent concentrations, which restricts site accessibility and extends the diffusion pathway for dye molecules.^31^ After reaching an adsorbent concentration of 27 mg L^−1^, the removal performance surpassed 94%, and further rises in the adsorbent concentration resulted in a minimal increase in removal efficiency.

#### 3.2.3. Effect of initial MB concentration

The adsorbent dosage is considered a crucial factor in the elimination of MB. The impact of various concentrations on MB elimination was examined, with the results illustrated in Fig. 5c. Fig. 5c illustrates that increasing the initial amount of LP from 2 mg L^−1^ to 100 mg L^−1^ markedly improved the adsorption capability of the RMI-5 composite from 20 mg g^−1^ to 198 mg g^−1^ despite a reduction in its elimination efficiency of LP from 96% to 91%. The increasing initial adsorbate amount boosts the mass transfer driving force by establishing a stronger concentration gradient between the adsorbent surface and the bulk solution.^32^ This process enhances the aggregation of additional adsorbate molecules per unit mass of adsorbent, thereby increasing adsorption capability. Conversely, at lower amounts, the ratio of accessible active sites to adsorbate molecules becomes higher, facilitating a greater proportion of the adsorbate to be adsorbed, thus enhancing removal efficiency. At elevated amounts, the limited number of adsorption sites becomes occupied more rapidly, resulting in a substantial amount of adsorbate remaining unabsorbed, which decreases removal efficiency.

#### 3.2.4. Adsorption kinetics

The effectiveness of an adsorbent in treating wastewater is assessed by its quick removal of adsorbates and rapid attainment of equilibrium within a short duration. Fig. 5d demonstrates the effect of adsorption time on elimination efficiency across various concentrations. In the early removal period (within 5 minutes of adsorption), the removal rate of RMI-5 for MB markedly increased by 87%. With an improvement in contact time, the removal rate exhibited a slow increase and ultimately stabilized after 60 minutes. This is a common phenomenon during the removal of pollutants, as the active sites on the adsorbent surface become saturated with an extended adsorption time.^33^ The removal equilibrium was attained within 60 minutes, achieving an elimination rate of 94%. Thus, RMI-5 demonstrated remarkably rapid adsorption rates and elevated removal efficiency.

The adsorption performance of the RMI-5 adsorbents was evaluated by applying the kinetic data to the intraparticle diffusion (IPD), pseudo-second-order (PSO), and pseudo-first-order (PFO) models. The equations for the PFO, PSO, and IPD kinetic models are, respectively, written as follows:

3 *q*_*t*_ = *q*_e_[1 − exp(*k*_1_*t*)],

4
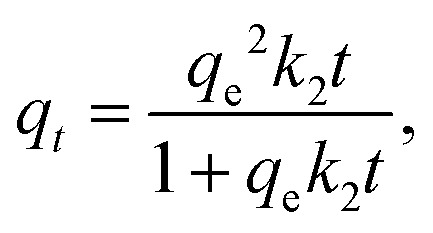

**Fig. 6:**
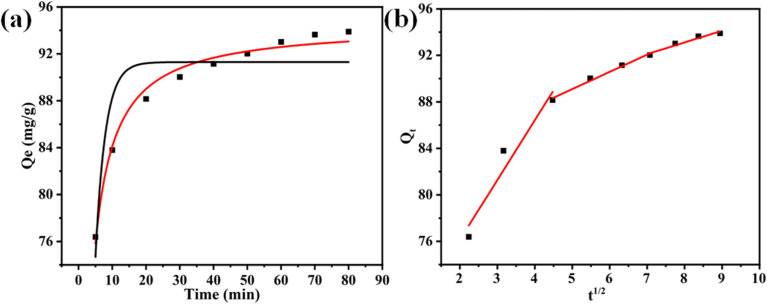
Kinetic model fit of MB dye: (a) red plot is for PSO and black is for PFO and (b) intraparticle diffusion model.

5 *q*_*t*_ = *k*_i_*t*^0.5^ + *C*,

where *q*_e_ denotes the removal capacity (mg g^−1^) of MB at equilibrium, *q*_*t*_ denotes the removal capacity (mg g^−1^) of MB at time *t*, *k*_1_ (min^−1^) and *k*_2_ (g mg^−1^ min^−1^) represent the rate constants of PFO, *k*_i_ represents the rate constant related to IPD, and *C* denotes the thickness of the boundary layer. The kinetic study indicated that the PSO model most accurately described the adsorption of MB onto RMI-5. The PSO kinetic model exhibited superior *R*^2^ values (0.986) compared to the PFO model (0.81) (Fig. 6a and Table S2), indicating that the adsorption of MB by the RMI-5 composite was governed by chemisorption.^34^

The uptake of MB on the RMI-5 composite was further analyzed using the intraparticle diffusion model to identify the rate-determining step (Fig. 6b). Two stages were detected in the IPD kinetic plots. MB was rapidly transferred from the liquid phase to the outer surface of the RMI-5 composite during the first stage *via* mass transfer or external diffusion. This phase is marked by a rapid rise in dye absorption, indicating the presence of active adsorption sites on the material's exterior surface.^35^ During the second phase, the adsorption rate diminishes as the dye molecules begin to diffuse into the interior pores of the composite. This process is regulated by intraparticle diffusion, which constitutes the rate-limiting phase following the saturation of the exterior surface. The multi-linear plots of the Weber–Morris model demonstrate that the adsorption process does not adhere to a singular path to the origin, signifying the participation of numerous stages throughout the process. The non-origin (*I* ≠ 0) intercepts indicate that adsorption is governed by many phases, which are characteristics of systems involving both exterior surface adsorption and internal pore diffusion. These findings underscore the significance of the exterior surface during the initial fast adsorption phase, which is succeeded by a more gradual intraparticle diffusion phase. This multi-phase process emphasizes the significance of surface and pore interactions in promoting effective dye removal, with internal diffusion gaining significance once the external surface sites are saturated.

#### 3.2.5. Adsorption isotherm

Freundlich and Langmuir isotherm models were employed to calculate the maximum removal capability and comprehend the underlying mechanism. The Langmuir model refers to monolayer adsorption, which is characterized by the absence of interactions between homogeneous active sites with identical energy levels and adsorbed contaminants. The Freundlich isothermal model refers to multi-layer adsorption, considering potential interactions between the heterogeneous distribution of adsorption energy and adsorbed contaminants. The equations for the Freundlich and Langmuir models are as follows:^36,37^6
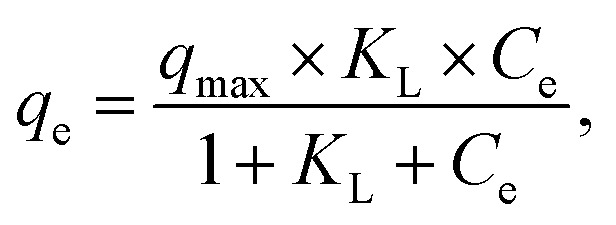

7 *q*_e_ = *K*_F_*C*^1/*nF*^,

where *q*_e_ represents the uptake capability (mg g^−1^) of MB at equilibrium, *C*_e_ denotes the amount of MB (mg L^−1^) at equilibrium, and *q*_max_ denotes the maximum uptake capacity of RMI-5 for MB. *K*_L_ and *K*_F_ denote the equilibrium constants of the Langmuir and Freundlich models, respectively.

**Fig. 7:**
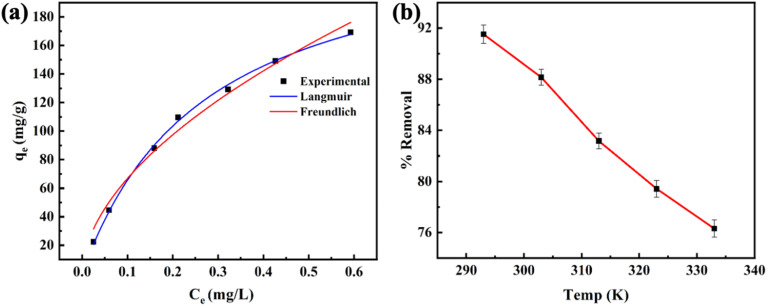
(a) Isotherm model fit of the MB dye and (b) the influence of temperature on the adsorption of MB.

The Langmuir model exhibited a higher adsorption capacity (245.2 mg g^−1^) and correlation coefficient (*R*^2^ > 0.997) than the Freundlich model (Fig. 7a and Table S3), suggesting that the adsorption of methylene blue on the RMI-5 composite surface is more consistent with the Langmuir model. This suggests that the removal of MB is homogeneous, resulting in a single layer on the RMI-5 composite with no interactions among the adsorbed molecules.

Additionally, the separation factor (*R*_L_), indicating the affinity of the removal procedure, was further examined. The equation for *R*_L_ is as follows.8
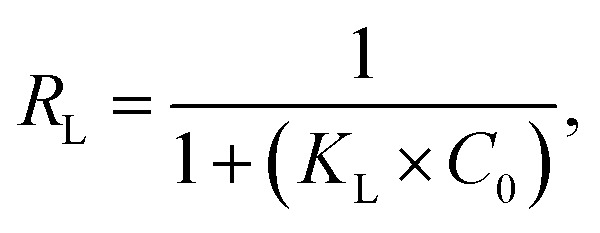
where *C*_0_ represents the initial amount (mg L^−1^) of the methylene blue, while *K*_L_ denotes the constant associated with the Langmuir model, *R*_L_ = 1 denotes linear adsorption, *R*_L_ > 1 denotes reverse adsorption, and 0 < *R*_L_ < 1 denotes favourable adsorption. For irreversible adsorption, *R*_L_ equals zero. *R*_L_ values ranged from 0 to 1, indicating a favourable adsorption behaviour of the adsorbents for MB. The adsorption effectiveness of RMI-5 for methylene blue was evaluated by comparing its elimination rate with that of previously documented adsorbents. Table 2 clearly illustrates that the synthesized RMI-5 demonstrates a much superior adsorption capacity (245.2 mg g^−1^) relative to most previously documented materials. The increased efficiency is due to NaI modification, which enhances the interaction between the adsorbent and methylene blue, thus illustrating the efficacy of the produced material. The enhanced performance of RMI-5 demonstrates the efficacy of this modification in augmenting adsorption efficiency, particularly under adjusted alkaline pH and ambient temperature conditions, rendering it a suitable material for dye removal applications.

**Table 2:** Comparison of the adsorption capacities of previously reported composites with RMI-5 for methylene blue

Adsorbent	Temperature (K)	pH	Adsorption capacity (mg g^−1^)	Ref.
MACz	298	4.9	156.25	38
Hyd/C30B	318	8	77.51	39
GO:CS	298	3–11	110.9	40
Fe-BDC MOF	303	4	23.92	41
Chitin/clay microspheres	313	1–11	156.7	42
MMTNS	313	8	91	43
Chitosan/κ-carrageenan/acid-activated bentonite	323	4	18.80	44
MSN-A	298	10	19.26	45
XG5	298	2.8	26.04	46
Sodium halide modified RM (RMI-5)	298	8	245.2	This study

#### 3.2.6. Effects of temperature and thermodynamic study

The adsorption studies of MNB on RMI-5 adsorbents were conducted at various temperatures to elucidate their thermodynamic characteristics. Fig. 7b illustrates the impact of temperature on the methylene blue elimination performance of the RMI-5. The adsorption performance of methylene blue decreased as the temperature increased, demonstrating the exothermic characteristics of the uptake process of MB.

Three thermodynamic variables, entropy change (Δ*S*°), enthalpy change (Δ*H*°), and Gibbs free energy (Δ*G*°), which govern energy transfer throughout the removal process and evaluate the feasibility of the adsorption process, were obtained:

9 Δ*G* = −*RT* ln *K*_D_,

10
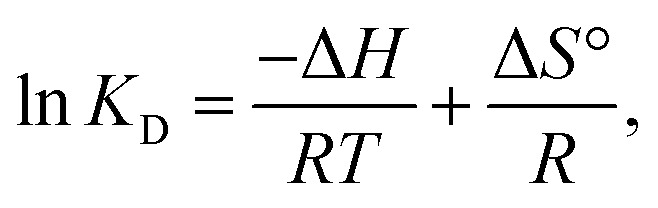
where *T* denotes temperature (K) and *R* represents the general gas constant, valued at 8.314 J mol^−1^ K^−1^. *K*_D_ represents the equilibrium parameter and was calculated by graphing ln *q*_e_/*C*_e_ against *q*_e_. All Δ*G*° values were negative at the tested temperatures, as demonstrated in Fig. S3 and Table S4, revealing that MB's adsorption behaviour on the RMI-5 adsorbents was both feasible and spontaneous. The negative values of the enthalpy change indicate the exothermic nature of the adsorption process of MB by the RMI-5 adsorbent. Furthermore, the MB molecules and adsorbents interacted through van der Waals interactions, hydrogen bonding, and electrostatic forces, as evidenced by the negative value of Δ*S*°.

#### 3.2.7. Adsorption mechanism

**Fig. 8:**
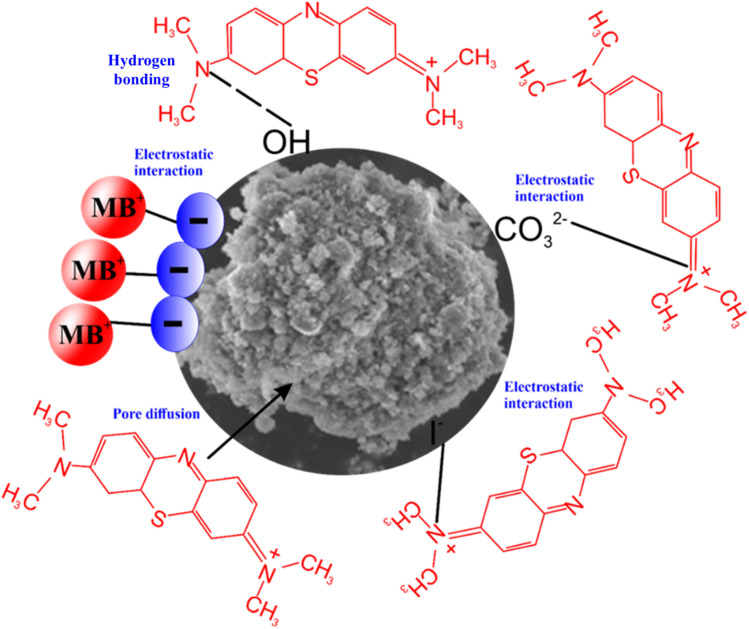
The possible mechanism for MB adsorption on the RMI-5 surface.

The adsorption mechanism of MB on the RMI-5 adsorbent surface was explored using FTIR. Fig. S4 compares RMI-5 fresh (before MB adsorption) and RMI-5 spent (after MB adsorption) to analyze the nature of the adsorption process. The OH peaks were displaced from 3440 cm^−1^ to 3404 cm^−1^ after the adsorption of MB, suggesting that the OH group was involved in the adsorption of MB. The absorption band at 1600 cm^−1^ is typical of C=C bonds. This likely indicates the presence of the benzene ring in the MB, showing that the MB was successfully adsorbed by RMI-5.^47^ The distinct peaks identified at 1415 and 992 cm^−1^, associated with the (CO_3_)^2−^ and O–Si–O groups, transformed to 1385 cm^−1^ and 1020 cm^−1^ after adsorption of MB dye, respectively. After capturing MB, the peaks detected at 556 cm^−1^, associated with the C–I group, were shifted to 542 cm^−1^. The peaks of the (CO_3_)^2−^, O–Si–O, and C–I groups shifted to lower wavenumbers after the MB molecules were adsorbed, indicating that these groups participated in MB adsorption. The proposed mechanism for methylene blue (MB) adsorption onto the RMI-5 composite is illustrated in Fig. 8. It involves three primary pathways: (a) hydrogen bonding: abundant hydroxyl (–OH) groups on the RMI-5 surface interact with the nitrogen atoms of MB molecules, forming strong hydrogen bonds that enhance dye retention. (b) Electrostatic attraction: the negatively charged surface of RMI-5, enriched by functional groups and anionic species, such as I^−^ and CO_3_^2−^, facilitates rapid adsorption of the cationic MB dye through strong electrostatic interactions. (c) Pore diffusion: the porous structure and high specific surface area of RMI-5 enable efficient dye molecule penetration and retention *via* intraparticle diffusion through micro- and mesopores. These synergistic mechanisms collectively contribute to the high adsorption efficiency observed. Overall, the findings highlight the significant potential of NaI-functionalized red mud as a cost-effective and high-performance adsorbent for the removal of MB dye from wastewater.

## 4. Conclusions

This study successfully demonstrated the synthesis of functionalized red mud (RM) composites using an impregnation method, resulting in a novel and efficient adsorbent for the removal of methylene blue (MB) from wastewater. The formation of hollow cores within the RM structure was effectively regulated by varying the NaI content during functionalization. Comprehensive characterization using XRD, DTG, TG, BET, and FTIR confirmed the enhanced thermal stability, surface properties, and chemical functionalities of the synthesized composites (RRM, RMI-1, RMI-3, and RMI-5). Among these, RMI-5 exhibited the highest adsorption capacity, achieving a maximum MB removal of 245.2 mg g^−1^ under optimal conditions. Adsorption kinetics and isotherm analyses revealed that the pseudo-second order and Langmuir models best described the process, indicating a chemisorption-driven monolayer adsorption mechanism. Thermodynamic parameters further supported that MB adsorption was spontaneous and exothermic. The predominant mechanisms involved included pore diffusion, hydrogen bonding, and electrostatic attraction. The NaI-modified red mud composite demonstrates considerable potential for the elimination of methylene blue and analogous contaminants from aqueous solutions. The findings indicate that this material may be utilized in extensive water purification systems, especially in sectors where dye-contaminated wastewater poses a significant issue.

## Author contributions

M. Sarfraz: supervision, writing – original draft, reviewing, and editing. F. Shafiq: project administration, writing – review & editing, supervision. I. Ijaz: writing – original draft, reviewing, and editing. I. A. Shaaban: data curation and methodology. N. Raza: formal analysis, investigation, and data curation. K. M. Albalawi: methodology and formal analysis. A. Waseem: data curation and investigation.

## Conflicts of interest

The authors declare that they have no known competing financial interests or personal relationships that could have appeared to influence the work reported in this paper.

## Supplementary Material

RA-015-D5RA06455D-s001

## Data Availability

Data will be made available on request. Supplementary information is available. See DOI: https://doi.org/10.1039/d5ra06455d.

## References

[cit1] Liu Y., Li B., Lei X., Liu S., Zhu H., Ding E., Ning P. (2022). Novel method for high-performance simultaneous removal of NO_x_ and SO_2_ by coupling yellow phosphorus emulsion with red mud. Chem. Eng. J..

[cit2] Lu J. Y., Bu Z. Q., Lei Y. Q., Wang D., He B., Wang J., Huang W. T. (2024). Facile microwave-assisted synthesis of Sb_2_O_3_-CuO nanocomposites for catalytic degradation of p-nitrophenol. J. Mol. Liq..

[cit3] Nadgire A. R., Barve S. B., Ithape P. K. (2020). Experimental investigation and performance analysis of double-basin solar still using CFD techniques. J. Inst. Eng. (India): Ser. C.

[cit4] Chowdhary P. , Bharagava R. N., Mishra S. and Khan N., Role of industries in water scarcity and its adverse effects on environment and human health, Environmental Concerns and Sustainable Development: Volume 1: Air, Water and Energy Resources, 2020, pp. 235–256

[cit5] Deng J., Wang H., Huang J., Hu J., Pan Z., Gao R., Wang L., Xu D. (2025). Synergistic TA/(NH_4_)_2_SO_4_ conditioning for sludge dewatering: Mechanisms of EPS redistribution and hydrophobic enhancement. J. Environ. Manage..

[cit6] He J., Tian G., Liao D., Li Z., Cui Y., Wei F., Zeng C., Zhang C. (2026). Mechanistic insights into methanol conversion and methanol-mediated tandem catalysis toward hydrocarbons. J. Energy Chem..

[cit7] dos Santos L. G., Machado L. F. L., Andrade L. S., Xavier G. T. M., Mandelli D., Carvalho W. A. (2025). Glycerol-based modified carbons as adsorbents for efficient and sustainable nitrate removal from wastewater. Energy Environ. Sustainability.

[cit8] Khan W. U., Ahmed S., Dhoble Y., Madhav S. (2023). A critical review of hazardous waste generation from textile industries and associated ecological impacts. J. Indian Chem. Soc..

[cit9] Fernandes M. , Fernandes R. D., Padrão J., Melro L., Alves C., Rodrigues R., Ribeiro A. I. and Zille A., Plasma in textile wastewater treatment, in Advances in Plasma Treatment of Textile Surfaces, Elsevier, 2024, pp. 267–322

[cit10] Boussaksou I., Aoulad El Hadj Ali Y., Azzouz A., Stitou M. (2024). Recent trends in biosorption: the removal of emerging dye pollutants from aqueous medium. Euro-Mediterr. J. Environ. Integr..

[cit11] Salleh M. A. M., Mahmoud D. K., Karim W. A. W. A., Idris A. (2011). Cationic and anionic dye adsorption by agricultural solid wastes: a comprehensive review. Desalination.

[cit12] Ismail M., Akhtar K., Khan M., Kamal T., Khan M. A., M Asiri A., Seo J., Khan S. B. (2019). Pollution, toxicity and carcinogenicity of organic dyes and their catalytic bio-remediation. Curr. Pharm. Des..

[cit13] Yildirim O. A., Bahadir M., Pehlivan E. (2022). Detrimental effects of commonly used textile dyes on the aquatic environment and human health – a review. Fresenius Environ. Bull..

[cit14] Hanafi M. F., Sapawe N. (2020). A review on the water problem associate with organic pollutants derived from phenol, methyl orange, and remazol brilliant blue dyes. Mater. Today: Proc..

[cit15] Samoylova N. A., Gureev A. P., Popov V. N. (2023). Methylene blue induces antioxidant defense and reparation of mitochondrial DNA in a Nrf2-dependent manner during cisplatin-induced renal toxicity. Int. J. Mol. Sci..

[cit16] Oladoye P. O., Ajiboye T. O., Omotola E. O., Oyewola O. J. (2022). Methylene blue dye: Toxicity and potential elimination technology from wastewater. Results
Eng..

[cit17] Li Y., Bu J., Sun Y., Huang Z., Zhu X., Li S., Chen P., Tang Y., He G., Zhong S. (2025). Efficient degradation of norfloxacin by synergistic activation of PMS with a three-dimensional electrocatalytic system based on Cu-MOF. Sep. Purif. Technol..

[cit18] Al-Gethami W., Qamar M. A., Shariq M., Alaghaz A.-N. M., Farhan A., Areshi A. A., Alnasir M. H. (2024). Emerging environmentally friendly bio-based nanocomposites for the efficient removal of dyes and micropollutants from wastewater by adsorption: a comprehensive review. RSC Adv..

[cit19] Lyu F., Niu S., Wang L., Liu R., Sun W., He D. (2021). Efficient removal of Pb(II) ions from aqueous solution by modified red mud. J. Hazard. Mater..

[cit20] Saveliev S., Yarosh T., Kondratenko M., Babaievska O., Baboshko D. Y. (2024). Current state and prospects of red mud utilisation: A review. IOP Conf. Ser.: Earth Environ. Sci..

[cit21] Zhou Y., Chen X., Peng Y., Chen Z., Chen X. (2025). Experimental study on construction application of red mud-based concrete pavement. Case Stud. Constr. Mater..

[cit22] Silveira N. C., Martins M. L., Bezerra A. C., Araújo F. G. (2021). Red mud from the aluminium industry: production, characteristics, and alternative applications in construction materials—a review. Sustainability.

[cit23] Wang L., Hu G., Lyu F., Yue T., Tang H., Han H., Yang Y., Liu R., Sun W. (2019). Application of red mud in wastewater treatment. Minerals.

[cit24] Zhang S., Cui J., Li Z., Huang H., Zhang W., Wang Z., Zhao F., Guo S. (2025). Waste reutilization: Carbon foam–red mud composites with photothermal effects for wastewater purification in outdoor environments. J. Environ. Manage..

[cit25] Li X., Dong R., Zhang R., Zhang Y., Lu P., Meng X., Li P. (2025). Performance improvement and the mechanisms of red mud oxygen carrier in chemical looping gasification using strontium doping strategy. Chem. Eng. J..

[cit26] Fan X., Li C., Zeng G., Zhang X., Tao S., Lu P., Tan Y., Luo D. (2012). Hg^0^ removal from simulated flue gas over CeO_2_/HZSM-5. Energy Fuels.

[cit27] Shoaib A. G., Van H.-T., Tran D.-T., El Sikaily A., Hassaan M. A., El Nemr A. (2024). Green algae Ulva lactuca-derived biochar-sulfur improves the adsorption of methylene blue from water. Sci. Rep..

[cit28] Liu J., Xie Y., Li C., Fang G., Chen Q., Ao X. (2020). Novel red mud/polyacrylic composites synthesized from red mud and its performance on cadmium removal from aqueous solution. J. Chem. Technol. Biotechnol..

[cit29] Pagnanelli F., Sambenedetto C., Furlani G., Vegliò F., Toro L. (2007). Preparation and characterisation of chemical manganese dioxide: Effect of the operating conditions. J. Power Sources.

[cit30] Al-Fakih A., Mohamed Nor Z., Inayath Basha S., Nasiruzzaman Shaikh M., Ahmad S., Al-Osta M. A., Aziz M. A. (2023). Characterization and applications of red mud, an aluminum industry waste material, in the construction and building industries, as well as catalysis. Chem. Rec..

[cit31] Yao Y., Xu F., Chen M., Xu Z., Zhu Z. (2010). Adsorption behavior of methylene blue on carbon nanotubes. Bioresour. Technol..

[cit32] Nautiyal P., Subramanian K., Dastidar M. (2016). Adsorptive removal of dye using biochar derived from residual algae after in-situ transesterification: alternate use of waste of biodiesel industry. J. Environ. Manage..

[cit33] Shafiq F., Liu C., Zhou H., Chen H., Yu S., Qiao W. (2023). Adsorption mechanism and synthesis of adjustable hollow hydroxyapatite spheres for efficient wastewater cationic dyes adsorption. Colloids Surf., A.

[cit34] Ijaz I., Bukhari A., Nazir A., Gilani E., Zain H., Shaheen A., Shaik M. R., Khan M. (2025). Synthesis of MoBT_x_@SF@PA composite for efficient and rapid adsorption of indomethacin and ceftriaxone: Unveiling exceptional adsorption performance and potential mechanisms. Sep. Purif. Technol..

[cit35] Ijaz I., Bukhari A., Nazir A., Gilani E., Zain H., Shaheen A., Shaik M. R., Khan M. (2025). Modification of bacterial cellulose by MoBT_x_ MBene and 1,4-dithiothreitol for rapid and efficient adsorption of indomethacin and losartan potassium. Int. J. Biol. Macromol..

[cit36] Wang X., Xu Q., Zhang L., Pei L., Xue H., Li Z. (2023). Adsorption of methylene blue and Congo red from aqueous solution on 3D MXene/carbon foam hybrid aerogels: A study by experimental and statistical physics modeling. J. Environ. Chem. Eng..

[cit37] Ijaz I., Bukhari A., Nazir A., Khan M., Gilani E., Zain H., Shaheen A., Hatshan M. R., Adil S. F. (2024). Functionalization of MXene using iota-carrageenan, maleic anhydride, and N,N′-methylene bis-acrylamide for high-performance removal of thorium (IV), uranium (IV), sulfamethoxazole, and levofloxacin. Int. J. Biol. Macromol..

[cit38] Yağmur H. K., Kaya İ. (2021). Synthesis and characterization of magnetic ZnCl_2_-activated carbon produced from coconut shell for the adsorption of methylene blue. J. Mol. Struct..

[cit39] Khatooni H., Peighambardoust S. J., Foroutan R., Mohammadi R., Ramavandi B. (2023). Adsorption of methylene blue using sodium carboxymethyl cellulose-g-poly(acrylamide-co-methacrylic acid)/Cloisite 30B nanocomposite hydrogel. J. Polym. Environ..

[cit40] Shi Y., Song G., Li A., Wang J., Wang H., Sun Y., Ding G. (2022). Graphene oxide-chitosan composite aerogel for adsorption of methyl orange and methylene blue: Effect of pH in single and binary systems. Colloids Surf., A.

[cit41] Soni S., Bajpai P., Mittal J., Arora C. (2020). Utilisation of cobalt doped Iron based MOF for enhanced removal and recovery of methylene blue dye from waste water. J. Mol. Liq..

[cit42] Xu R., Mao J., Peng N., Luo X., Chang C. (2018). Chitin/clay microspheres with hierarchical architecture for highly efficient removal of organic dyes. Carbohydr. Polym..

[cit43] Wang W., Zhao Y., Bai H., Zhang T., Ibarra-Galvan V., Song S. (2018). Methylene blue removal from water using the hydrogel beads of poly (vinyl alcohol)-sodium alginate-chitosan-montmorillonite. Carbohydr. Polym..

[cit44] Ulu A., Alpaslan M., Gultek A., Ates B. (2022). Physics, Eco-friendly chitosan/κ-carrageenan membranes reinforced with activated bentonite for adsorption of methylene blue. Mater. Chem. Phys..

[cit45] Usgodaarachchi L., Thambiliyagodage C., Wijesekera R., Bakker M. G. (2021). Synthesis of mesoporous silica nanoparticles derived from rice husk and surface-controlled amine functionalization for efficient adsorption of methylene blue from aqueous solution. Curr. Res. Green Sustainable Chem..

[cit46] Seera S. D. K., Kundu D., Gami P., Naik P. K., Banerjee T. (2021). Synthesis and characterization of xylan-gelatin cross-linked reusable hydrogel for the adsorption of methylene blue. Carbohydr. Polym..

[cit47] Li Y., Liu Z., Wan X., Xie L., Chen H., Qu G., Zhang H., Zhang Y.-F., Zhao S. (2023). Selective adsorption and separation of methylene blue by facily preparable xanthan gum/amantadine composites. Int. J. Biol. Macromol..

