# Supplementary material for: Sodium iodide modified-red mud for efficient adsorptive removal of methylene blue from wastewater: isotherm modeling and adsorption
Source: RSC Adv. 2025 Oct 24;15(48):40456–68. doi: 10.1039/d5ra06455d (PMC12550888; doi:10.1039/d5ra06455d)
Supplement: RA-015-D5RA06455D-s001 [file RA-015-D5RA06455D-s001.pdf]

Supplementary Information (SI) for RSC Advances.
This journal is © The Royal Society of Chemistry 2025

SUPPLEMENTARY INFORMATION

Sodium Iodide Modified Red Mud for Efficient Adsorptive Removal of Methylene Blue from Wastewater: Isotherm modeling and adsorption

Muhammad Sarfraz[a], Farishta Shafiq[a], Karma M Albalawi[b], Nadeem Raza[c], Ibrahim A. Shaaban[d], Asim Waseem[a], Irfan Ijaz[e]*,

[a] State Key Laboratory of Fine Chemicals, School of Chemical Engineering, Dalian University of Technology, Dalian, 116024, P. R. China
[b]Department of Chemistry, Faculty of Science, University of Tabuk, Tabuk, Saudi Arabia
[c] Department of Chemistry, College of Science, Imam Mohammad Ibn Saud Islamic University (IMSIU), Riyadh, KSA
[d]Research Center for Advanced Materials Science (RCAMS), Chemistry Department, Faculty of Science, King Khalid University, P.O. Box 9004, Abha 61413, Saudi Arabia
[e]School of Chemistry, Faculty of Basic Sciences and Mathematics, Minhaj University Lahore, Lahore 54700, Pakistan

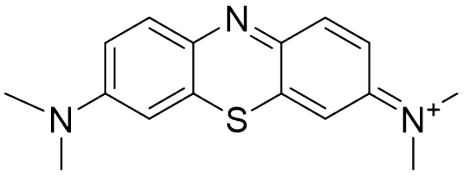

Fig. S1. Chemical structure of methylene blue.

Table S1. The chemical composition of different buffers used to maintain the pH.

Sr. No.	pH	Buffer composition	Final volume / mL
1	2	25 ml KCl (0.2 M) + 6.6 ml HCl (0.2 M)	
2	3	50 ml KHP (0.1 M) + 22.5 ml HCl (0.1 M)	
3	4	50 ml KHP (0.1 M) + 2 ml NaOH (0.1 M)	
4	5	50 ml KHP (0.1 M) + 22.5 ml NaOH (0.1 M)	
5	6	50 ml KH_2PO_4 (0.1 M) + 5.8 ml NaOH (0.1 M)	100
6	7	50 ml KH_2PO_4 (0.1 M) + 29 ml NaOH (0.1 M)	
7	8	50 ml KH_2PO_4 (0.1 M) + 46.1 ml NaOH (0.1 M)	
8	9	50 ml Borax (0.025 M) + 4.7 ml NaOH (0.1 M)	
9	10	50 ml $NaHCO_3$ (0.05 M) + 10.8 ml NaOH (0.1 M)	
10	11	50 ml $NaHCO_3$ (0.05 M) + 22.8 ml NaOH (0.1 M)	

Validation of Beer-Lambert law

As demonstrated in Fig. S2 the Beer-Lambert law was used to validate the linearity of the UV-Vis response at 668 nm for MB.

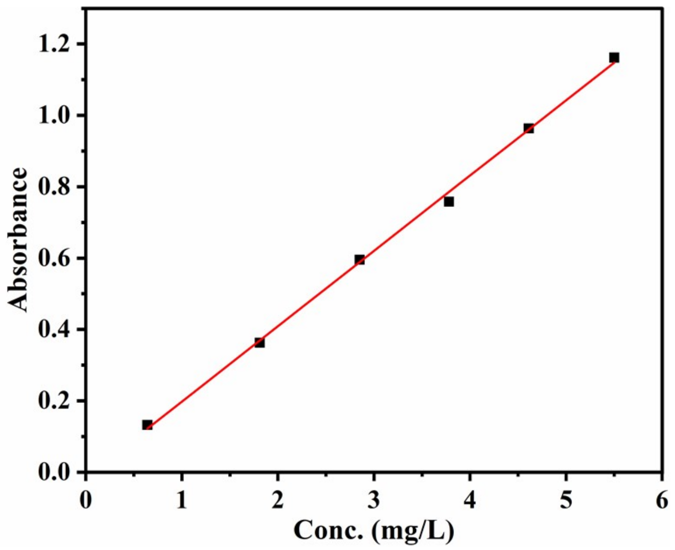

Fig. S2. Beer-Lambert law validation for MB at λ = 664 nm.

Solutions of different concentrations were prepared by dilution of a 1000 mg/L stock solution. Each solution's absorption spectrum was obtained at 668 nm. The graph of absorbance vs concentration yielded an R^2 value of 0.99, indicating that MB has a strong linear response. The intensity of absorption rises with dye concentration due to increased contact between dye-dye molecules, as proven by the positive value of R^2.

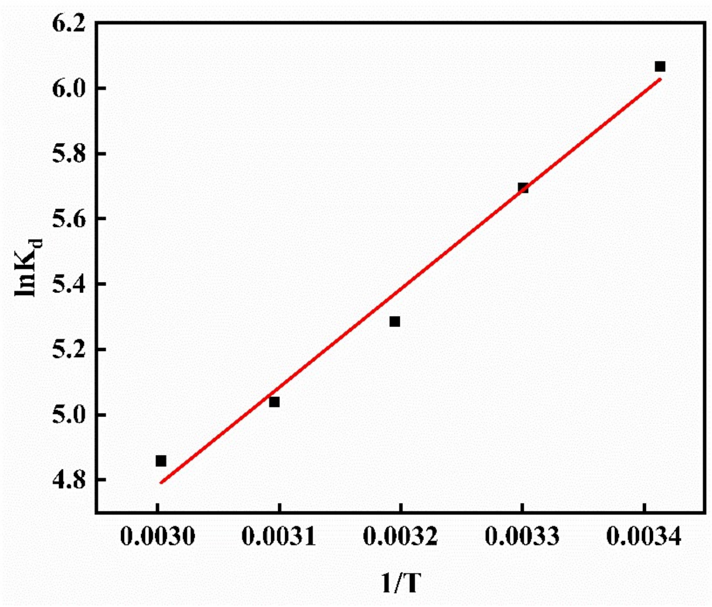

Fig. S3. Van't Hoff plot for adsorption of MB dye on RMI-5 adsorbent.

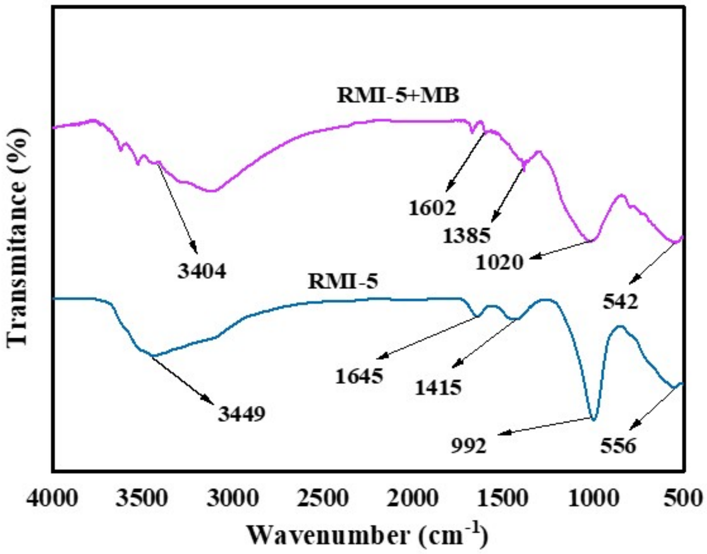

Fig. S4. The FTIR spectra before and after adsorption of MB.

The graph in Figure S5 presents the MB removal capacities of four different adsorbents: RRM, RMI-1, RMI-3, and RMI-5. The adsorption capacity is represented as qe (mg/g), showing how much MB dye is removed per gram of adsorbent. As seen in the graph, RMI-5 exhibits the highest MB removal capacity, reaching approximately 250 mg/g, followed by RMI-3 and RMI-1 with lower capacities. RRM shows the lowest adsorption efficiency. This data highlights the increased effectiveness of RMI-5 in comparison to the other adsorbents, demonstrating the superior performance of the NaI-modified red mud composite.

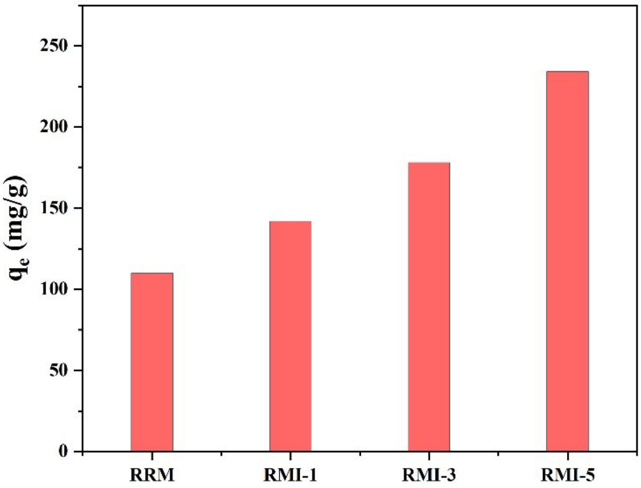

Fig. S5. Comparison of the methylene blue (MB) adsorption capacities (q_e, mg/g) of different adsorbents: RRM, RMI-1, RMI-3, and RMI-5.

Table S2 Kinetic parameters for the removal of MB by RMI-5

Models	Parameters	MB Data
**Pseudo-first order**	q_e (mg/g)	91.296
	k_1 (min^{-1})	0.3407
	R^2	0.81
**Pseudo-second order**	q_e (mg/g)	94.48
	k_2 (g (mg min)$^{-1}$	8.59×10^{-3}
	R^2	0.986
**Intra-particle diffusion**	k_i (mg/(g min ^{½}))	0.7
	I (mg/g)	87.4
	R^2	0.95
	k_i (mg/(g min ^{½}))	1.4
	I (mg/g)	81.8
	R^2	0.99
	k_i (mg/(g min ^{½}))	5.1
	I (mg/g)	65.8
	R^2	0.94

Table S3 Adsorption isotherm model parameters for MB adsorption by RMI-5

Models	Parameters	MB Data
**Langmuir**	R^2	0.997
	R_L	0.4849-0.8840 (favorable)
	q_m (mg/g)	245.2
	K_L (L/mg)	3.65
**Freundlich**	R^2	0.9822
	N	0.5452
	K_f (mg/g) (mg/L) $^{-1/n}$	234.3135

Table S4 Thermodynamic parameters for MB adsorption on the RMI-5 surface

Dye	Temperature (K)	ΔG° (kJ/mol)	ΔH° (kJ/mol)	ΔS° (kJ/mol)
MB	293	-17.57	-33.63	-0.054
	303	-17.03		
	313	-16.48		
	323	-15.93		
	333	-15.38		
